# Challenging FRET-based E-Cadherin force measurements in *Drosophila*

**DOI:** 10.1038/s41598-017-14136-y

**Published:** 2017-10-20

**Authors:** Dominik Eder, Konrad Basler, Christof M. Aegerter

**Affiliations:** 1 Institute of Molecular Life Sciences, University of Zurich, Zurich, CH-8057 Switzerland; 2 Institute of Physics, University of Zurich, Zurich, CH-8057 Switzerland

## Abstract

Mechanical forces play a critical role during embryonic development. Cellular and tissue wide forces direct cell migration, drive tissue morphogenesis and regulate organ growth. Despite the relevance of mechanics for these processes, our knowledge of the dynamics of mechanical forces in living tissues remains scarce. Recent studies have tried to address this problem with the development of tension sensors based on Förster resonance energy transfer (FRET). These sensors are integrated into force bearing proteins and allow the measurement of mechanical tensions on subcellular structures. Here, we developed such a FRET-based sensor to measure E-Cadherin tensions in different *Drosophila* tissues *in* and *ex vivo*. Similar to previous studies, we integrated the sensor module into E-cadherin. We assessed the sensitivity of the sensor by measuring dynamic, developmental processes and mechanical modifications in three *Drosophila* tissues: the wing imaginal disc, the amnioserosa cells and the migrating border cells. However, these assays revealed that the sensor is not functional to measure the magnitude of tensions occurring in any of the three tissues. Moreover, we encountered technical problems with the measurement of FRET, which might represent more general pitfalls with FRET sensors in living tissues. These insights will help future studies to better design and control mechano-sensing experiments.

## Introduction

Every living cell is embedded in a 3D- microenvironment where it is exposed to a variety of mechanical cues. It is getting more and more clear that – apart from biochemical cues – the physical parameters from the cellular environment strongly influence cellular behavior. Cells harbor machinery allowing them to sense and respond to these mechanical cues thereby ensuring their survival and the maintenance of tissue integrity and function. *In vitro* studies on single cells revealed that mechanical cues regulate cell migration^1^, cell differentiation^2,3^, the orientation and rate of cell division^4,5^ and the activation of signaling pathways^6^. In multicellular culture systems mechanics also influenced growth^7–9^ and migration^10^.

Advances in image acquisition techniques allowing the tracking of tissue dynamics revealed the relevance of mechanical cues not only for *in vitro* systems, but also for the development of living tissues. Tissue mechanics has been shown to alter cell mobility and the orientation of division plane during gastrulation in zebrafish, *Drosophila* and *C. elegans* ^11–13^. For the *Drosophila* wing imaginal disc, a well-established model for growth regulation - computational growth models^14–17^ and mechanical stimulation experiments^18^ suggested a key role of mechanical forces for growth and size regulation.

Despite increasing interest and technical advancements in the field of biomechanics, the measurement and quantification of mechanical quantities in living tissues remains challenging. The techniques most commonly used for *in vitro* studies (reviewed in^19,20^) are not applicable *in* and *ex vivo*: they either rely on direct contact with the structure to measure force – which is mostly impossible for living tissues - or the measurement has a time resolution that is not appropriate for living processes.

Therefore, imaging-based methods such as laser-ablation and force inference are most convenient to monitor physical properties in living tissues. For laser-ablation, a cellular structure is ablated with a focused laser beam to probe the tension state before the cut. In the *Drosophila* wing disc, laser ablation has provided insights into the distribution of tensions throughout the tissue^21,22^. However, the invasiveness of laser ablation makes it unsuitable for measuring dynamic processes over time. Force inference, a non-invasive, computational tool, determines edge tensions and internal cellular pressure by analyzing cell shapes. Force inference greatly depends on prior assumptions of mechanical equilibrium, force balance and homogeneous mechanical properties. Hence, it requires further validation of its results with other methods. A promising alternative are FRET (Förster Resonance Energy Transfer)- based tension sensors. These sensor modules usually consist of two fluorophores linked with an elastic spacer. The FRET efficiency provides a measure for the tension exerted onto the sensor module^23,24^. Such sensors have already been used to measure tensions over proteins which are expected to be involved in mechanotransduction, e.g. Vinculin, Talin or E-Cadherin^23–27^.

Here, we generated a FRET-based sensor for use in various tissues in *Drosophila melanogaster*. To address the role of mechanical tensions at the cell-cell contacts during development, we integrated a FRET module into the adherens junction protein E-Cadherin. To assess the functionality of our sensor, we measured FRET values in the wing imaginal disc, the amnioserosa cells and the border cells. To our surprise, the FRET values neither represented the expected tension patterns, nor responded to mechanical manipulations. Hence, the FRET module was not sensitive to mechanical forces in these *Drosophila* tissues. This work reveals the technical challenges of FRET tension sensors and highlights common pitfalls with the interpretation of FRET results, especially in dense, living tissues.

## Results

### Development of a new E-Cadherin tension sensor

**Figure 1:**
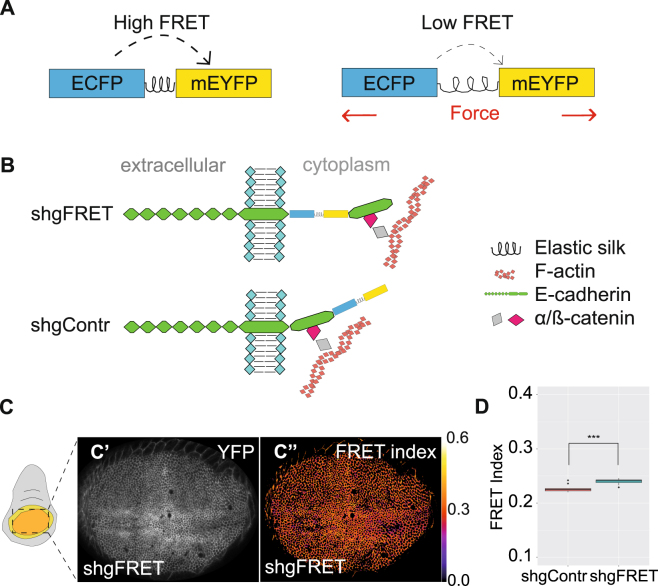
Developing a FRET tension sensor for E-Cadherin. (**A**) The tension sensor consists of ECFP and mEYFP connected by an elastic linker (GPGGA)_8_. FRET efficiency is high in a relaxed state but should decrease if external forces extend the sensor module. (**B**) The sensor module was integrated into the cytoplasmic domain of E-Cadherin adjacent to the transmembrane domain (shgFRET) to measure forces along the protein. The sensor module was also attached at the C-terminus of E-Cadherin (shgContr) lying outside of the force transducing domain to serve as a zero-force control. (**C**) YFP expression (C’) and corresponding FRET index (C”) of shgFRET shows that FRET was detectable in the wing pouch. (**D**) FRET index in the wing pouch of shgContr (0.227, n = 10) was significantly lower than for shgFRET (0.240, n = 10).

It is widely accepted that mechanical forces are propagated across an epithelial tissue from cell to cell via the adherens junction complex (reviewed in^28–30^). According to the current model, the transmembrane protein E-Cadherin forms homophilic bonds with E-Cadherins from adjacent cells whereas the cytoplasmic domain recruits α- and β- catenins which in turn associate with F-actin. Hence, E-Cadherin physically links neighboring cells to the cytoskeleton and is likely an appropriate protein to measure mechanical forces across epithelial tissues. We designed a tension sensor based on FRET in a fashion similar to the well- establised TSMod sensor^23^. Our sensor cassette consisted of ECFP and mEYFP which were connected by an elastic linker (GPGGA)_8_ derived from spider silk (Fig. 1A). If the tension on the sensor is low, the two fluorophores are close enough to allow for FRET. With increased tension, the distance between the fluorophores increases and the FRET efficiency decreases. Hence, the FRET efficiency should correlate with the tension across the sensor.

We inserted the sensor cassette into the cytoplasmic domain of E-Cadherin, between the transmembrane domain and the β- catenin binding domain (shgFRET) (Fig. 1B). Additionally, we generated a control construct in which the sensor cassette was attached at the C-terminus, which lies outside of the force transmitting region (shgContr) (Fig. 1B). This construct should control for any FRET influencing effect other than the mechanical forces across the protein, such as conformational changes, molecular crowding, etc. With these constructs we generated transgenic flies by a *knock-in* into the endogenous *shotgun* locus. Hence, the sensor was integrated into the endogenous E-Cadherin, and in homozygous flies no other E-Cadherin interfered with the measurements. Flies homozygous for shgFRET or shgContr were fertile and viable without any obvious phenotype, indicating that the constructs were fully functional. In addition to these sensor constructs, we also tested a very similar E-Cadherin sensor, Cad^TS^, which was developed previously^27^. Instead of being under endogenous regulation such as in shgFRET, Cad^TS^ is under the control of the *ubiquitin-* promoter.

In order to measure FRET, we developed a workflow including confocal microscopy and image processing. We used the ratiometric method to calculate the FRET index by detecting sensitized emission, which partially corrects for variability in confocal image acquisition (ref.^31^, see Material and Methods). It is important to consider that this method is very dependent on image acquisition parameters and therefore susceptible to imaging artifacts. To test whether this workflow is applicable to measure FRET in various *Drosophila* tissues, we used an established ATP (Adenosine triphosphate) FRET sensor as a positive control^32^. We were able to reproduce the published results in the salivary gland, the wing disc and the border cells (Fig. S1). As a proof of principle we measured FRET indices of shgFRET and shgContr in the *Drosophila* wing imaginal disc showing that FRET is taking place when the sensor was expressed in the wing disc (Fig. 1C). The average FRET index in the wing pouch region of the wing disc was higher for shgFRET than shgContr (Fig. 1D). To exclude that intermolecular FRET takes place between fluorophores from neighboring molecules, we looked at wing discs expressing E-Cadherins with only donor and only acceptor in parallel. Intermolecular FRET was not detectable in these wing discs (Fig. S2A).

### FRET measurements in the wing disc

**Figure 2:**
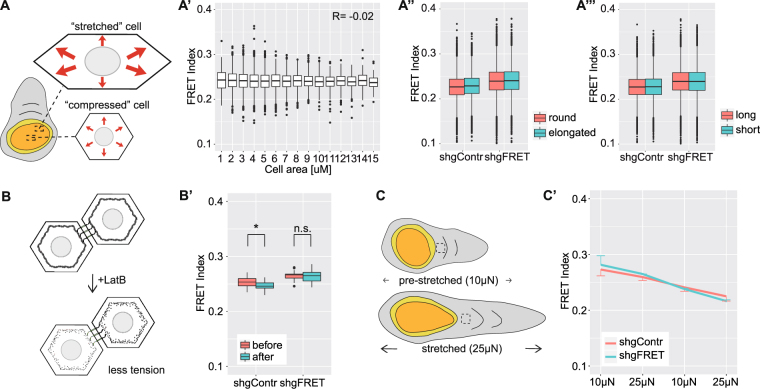
Single cell analysis and functional experiments in the wing disc. (**A**) Schematic drawing illustrates that cells in the center of the pouch are small, round and supposed to be mechanically compressed, whereas marginal cells are larger, elongated and mechanically stretched. In the marginal cells, the short edges are exposed to more mechanical stress than the long edges. These assumptions lead to following comparisons: (A’) The FRET index did not correlate with cell area in the wing pouch (here shown for shgFRET, R represents the Pearson correlation coefficient). (A”) The FRET index did not differ between long and short edges for shgContr (0.228 vs. 0,227) and shgFRET (0.240 vs. 0.240). (A”’) The FRET index did not differ between round and elongated cells for shgContr (0.229 vs. 0.226) and shgFRET (0.240 vs. 0.240). (Data for A’, A” and A”’ were pooled from 14 wing discs; n > 17.000 cells, n > 40.000 edges). (**B**) LatrunculinB treatment reduces cortical tension. (B’) FRET index decreased for shgContr (2.7%, n = 18) and shgFRET(0.4%, n = 18) upon treatment within 5 minutes. (detailed figure in Fig. S2C) (**C**) Using a stretching device, a pre-stretched (applied force of 10µN) and a stretched (25 µN) wind disc were compared. The dashed rectangle indicates the analyzed area. (C’) When cyclically altered between the two states every 5 minutes, a strong decay over time was observed for shgContr (18%, n = 2) and shgFRET (23%, n = 2), but no impact of the force change.

In order to evaluate the sensors functionality in the wing disc, we tested whether FRET distributions mirror the tension patterns across the wing pouch. It has been shown previously, that cells in the center of the wing pouch are mechanically compressed, whereas cells at the periphery are circumferentially stretched^15,21,22,33,34^. Heat maps of FRET distributions in the wing disc did not reveal any obvious pattern (Fig. 1C), so we further analyzed the results in more detail. It was shown before that the stretched cells were larger and more elongated than the compressed cells^15,22^ (Fig. 2A). However, the FRET indices did not correlate with cell size in the wing pouch of shgFRET and shgContr flies (Fig. 2A’, Fig. S2B). Further, we distinguished the cells of the wing pouch by shape between round and elongated cells, because we expected the elongated ones to be stretched (Fig. 2A”). However, FRET indices did not differ between round and elongated cells. It could be possible that an effect averaged out because the shorter edges of a stretched cell were under higher perpendicular tension than the long edges. But FRET indices also did not vary between the long and short edges of the cells (Fig. 2A”’). Thus, by analyzing the FRET index distribution of our sensor lines, we could not detect any evidence of the global tension patterns reported in the wing pouch.

Further, we performed manipulations to experimentally modify the tension on E-Cadherin. We decreased the cortical tension in the wing disc cells by LatrunculinB treatment, which effectively inhibits actin polymerization (Fig. 2B, Fig. S3A,B). Instead of an increase in FRET index due to LatrunculinB treatment, we observed a decay of the FRET indices in the shgFRET and the shgContr discs (Fig. 2B’). Having a negative control without treatment revealed that FRET indices decrease over time in culture even without any treatment or manipulation (Fig. S2C). We observed also in other experiments that the FRET indices decay over time in tissue culture, which is a more general “culturing artifact” in our setup. We thus staged all experiments precisely in time and always added controls without treatment to monitor the time-dependent decay. However, the effect of LatrunculinB treatment on shgFRET did not differ from the negative controls, which indicates a more general effect rather than a tension-specific one.

Because shgFRET did not react to a decrease in tension, we applied an external tensional force to the entire wing disc to increase the tension across the cells. For this we used a previously developed stretching setup which allowed us to stretch the cultured wing disc longitudinally with a defined force (Fig. 2C,^18^). We measured the FRET index at two different forces: 10 µN (pre-stretched) and 25 µN (stretched). Alternating between these two states did not affect the FRET index in the hinge region (Fig. 2C’). We again only observed a time-dependent decrease of FRET for shgFRET and shgContr.

Additionally, we experimentally increased tension by applying an osmotic shock with distilled water. Again, shgFRET and shgContr were affected similarly, indicating a force-independent effect (Fig. S2E–H).

Thus, not only did the distribution of FRET across the wing disc not resemble the reported patterns of mechanical tensions that have been described earlier, but direct mechanical manipulations only altered the FRET index of shgFRET to the same extent as for the negative control shgContr. This was also true when we repeated the experiments with Cad^TS^, the sensor that has previously been shown to be functional in border cell migration (ref.^27^, Fig. S5). This indicates that changes in FRET index are directly influenced by the experimental procedure rather than specifically by mechanical tensions in the wing disc.

### FRET measurements in the amnioserosa cells

**Figure 3:**
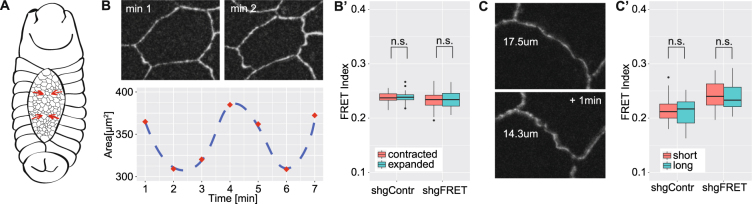
FRET analysis of the amnioserosa cells during dorsal closure. (**A**) In the early *Drosophila* embryo, the dorsal gap of the epidermis is closed by contractions of the underlying amnioserosa cells. (**B**) Amnioserosa cells undergo cycles of contraction and relaxation taking around four minutes. Here we show an example of one cell with measured values (red) and a guide to the eye (blue). (B’) FRET index did not significantly differ between contracted and expanded cells for shgContr (0.239 vs. 0. 239, n = 24) and shgFRET (0.233 vs. 0.235, n = 26). (**C**) If two neighboring cells contract, the conjunctive edge also contracts. (C’) For edges that contracted or expanded within one minute, the FRET index did not significantly differ between the shortened and elongated state for shgContr (0.216 vs. 0. 214, n = 31) and shgFRET (0.243 vs. 0.241, n = 29).

In the wing disc, mechanical tensions build up due to tissue growth and are therefore changing over long time scales. In contrast, in the amnioserosa cells during dorsal closure, mechanical tensions are highly dynamic. The amnioserosa cells underlying the dorsal gap undergo rapid waves of contraction and expansion on the time scale of minutes (Fig. 3A,B). These pulses are driven by the actomyosin cytoskeleton and pull the surrounding epidermal cells to subsequently close the dorsal opening^35–37^. E-Cadherin is very likely required for the transmission of the forces generated during dorsal closure^38,39^.

Therefore, we expect that FRET values of our tension sensor would decrease in a contracting amnioserosa cell and increase if the cells are expanding. But instead of a decrease in FRET index upon contraction, there was no significant difference between contracted and expanded cells (Fig. 3B’). A problem of the analysis of single cells was that we could not discriminate between E-Cadherin from two neighboring cells which share an edge. Thus, also the pulsing stage of the neighboring cells influenced the analysis, which could have averaged out an effect.

Therefore, we analyzed single edges and sought for edges that either contract or expand within one minute (Fig. 3C). Thereby, we could ascertain that in this specific region forces are generated and that at both sides of the edge the force is propagated. But also with this type of analysis, we did not see a response of the tension sensor to the changed mechanical state of the edge (Fig. 3C’).

To conclude, in the amnioserosa cells it is known that forces are generated and change cyclically over short time-scales, which can be observed by shape changes of the cells and their edges. However, neither our tension sensor, nor the published sensor Cad^TS^ (Fig. S6), did respond to these dynamics and FRET values did not change accordingly.

### FRET measurements of border cell migration

**Figure 4:**
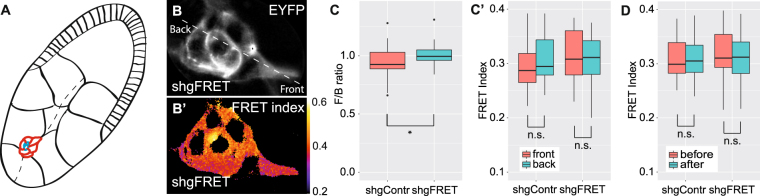
FRET analysis of border cell migration. (**A**) During stage 9 in the egg chamber, a cluster of six to eight border cells (red) and two polar cells (blue) migrate from the anterior pole through the nurse cells towards the oocyte (path as dashed line). (**B**) Border cell cluster forms protrusions at the leading cell to navigate between the nurse cells. FRET index was detectable in the entire cluster, here illustrated in the YFP channel and the corresponding FRET index. (**C**) The relative front to back ratio of the FRET index was significantly lower for shgContr (0.95, n = 40) than for shgFRET (1.01, n = 38). (C’) But the absolute values between front and back did not differ significantly for shgContr (0.294 vs. 0.310, n = 40) nor for shgFRET (0.313 vs. 0.311, n = 38). (**D**) The release of tension upon the treatment with the myosin blocker Y-27632 did not significantly change the FRET index for shgContr (0.312 vs. 0.317, n = 40) nor for shgFRET (0.320 vs. 0.313, n = 38).

Border cells of the *Drosophila* ovary have emerged as a model system for collective cell migration^40^. The border cells constitute a cell cluster, which detach from the anterior follicular epithelium at stage 9 of the egg chamber, and subsequently migrate posterior towards the oocyte (Fig. 4A). E-cadherin is required for border cell migration and especially for direction sensing of the cluster^27,41^. It was reported that E-cadherin is under higher tension in the front of the cluster, compared to the back of the cluster, which leads to a persistent and directed movement of the cluster^27^.

As tension of E-cadherin is supposed to be higher in the front, the FRET index should be lower. Therefore we calculated the front to back ratio by choosing small areas of around 20 µm^2^ in the leading and the rear cell. The ratio of the shgFRET sensor does not reveal any difference in FRET index between front and back. In contrast, the shgContr line had a significantly lower front to back ratio than shgFRET (Fig. 4C). Therefore, we looked at the absolute values of FRET indices, where no significant differences between the cells in the front and the back of the cluster were detectable, neither for shgFRET nor for shgContr (Fig. 4C’).

We further asked whether the FRET index of our sensor represents at all mechanical tensions across E-cadherin in the border cell. We performed a treatment with the Rho kinase inhibitor Y-27632, which inhibits myosin activity and indirectly reduces the tension on E-cadherin^27^. But for shgFRET and shgContr the treatment with Y-27632 did not have a significant effect on the FRET index (Fig. 4D).

To conclude, neither the shgFRET sensor and surprisingly not even the Cad^TS^ sensor (Fig. S6) did report the expected difference of mechanical tension between the front and the back of the cell cluster. Because inhibiting myosin did not affect the sensor, we concluded that the FRET values that we measured did not represent mechanical tensions during border cell migration.

### Fluorescence lifetime imaging microscopy to measure FRET efficiency

In order to test whether the negative outcome of the functional tests is due to a lower sensitivity of the ratiometric method, we repeated the experiments with Fluorescence Lifetime Imaging Microscopy (FLIM), an alternative method of FRET determination. FLIM is based on the fact that every fluorophore has a characteristic lifetime, which is the average time between the excitation and the emission of fluorescence. The lifetime of a fluorophore is sensitive to its molecular environment, which includes FRET dependent quenching. Hence, the lifetime is a direct read-out for FRET^31,42–44^. There are two main advantages of FLIM over the ratiometric methods: (1) Because the lifetime is independent of the fluorescence intensity, FLIM is less susceptible to imaging artifacts and therefore has a much better signal to noise ratio – or in short, it is a more sensitive measure of FRET. (2) In biological tissues, the ratiometric method remains semi-quantitative and provides only a relative value of FRET. With FLIM we obtain the absolute FRET efficiency, which allows comparing values between different experiments with different settings.

**Figure 5:**
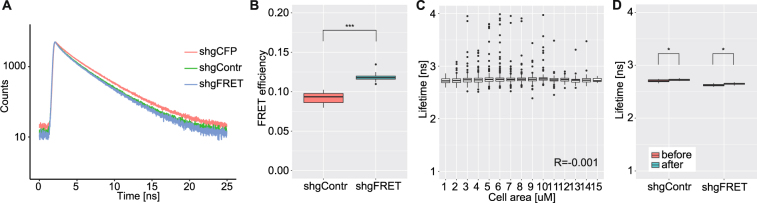
FLIM measurements in the wing disc. (**A**) Fluorescence decay curves of shgContr and shgFRET were almost overlapping, and both revealed lower lifetimes than shgCFP (n = 5). (**B**) Calculated FRET efficiency was significantly lower for shgContr (0.09, n = 15) than for shgFRET (0.12, n = 14). (Corresponding lifetimes are shown in Fig. S4B) (**C**) Lifetimes of single cells in the wing pouch did not correlate with cell size (here for shgFRET, pooled from 8 wing discs, n > 5000 cells, R represents Pearson correlation coefficient). (**D**) Lowering tensions by LatrunculinB treatment similarly increased lifetimes of shgContr and shgFRET for 1% (n = 9).

For technical reasons, we only measured FLIM in the wing disc. The divergence of the fluorescence decay curves of shgCFP (a sample which only included the donor and not the acceptor) with shgFRET and shgContr confirmed that FRET takes place between the donor and the acceptor (Fig. 5A). The calculated FRET efficiency was higher in shgFRET compared to shgContr (Fig. 5B, Fig. S4B). This was in agreement with our data from the ratiometric method, but somewhat contradicted the design of the sensor, where the zero-force control shgContr was expected to have higher FRET due to lower tension. Furthermore, we tested whether lifetimes correlated with the size of the cells because size depends on the tension of a cell. But no correlation between lifetime and cell size was detectable (Fig. 5C, Fig. S4A). Neither did the shape of a cell or the length and orientation of an edge affect the lifetimes (Fig. S4E,F). When performing LatrunculinB treatment, we observed the same effect as with the ratiometric method (Fig. 2B): the lifetimes of shgContr and shgFRET both increased and hence the FRET efficiency decreased (Fig. 5D). Time controls for shgFRET and shgContr without treatment had the same decrease of FRET over time as the treated samples, indicating a more general, tension-independent effect of culturing the wing disc *ex vivo* (Fig. S4C). With this we confirmed that the above described “culturing artifact” is not a measuring artifact from the ratiometric method but that culturing the wing disc indeed affects the FRET efficiency over time (Fig. S4C). Also the treatment with distilled water had an effect on the FRET efficiencies of both, shgContr and shgFRET, indicating that this is a force-independent effect (Fig. S4D).

Together, the data obtained from FLIM measurements confirmed the results from the ratiometric method and did not reveal any force-specific effect in the wing disc. Surprisingly, the FRET efficiencies of shgFRET were higher than the ones of shgContr, which was not in accordance with the idea that shgContr presents a zero-force control in which the fluorophores should be closer together and the FRET efficiency therefore higher.

## Discussion

As the results presented above show, the FRET-based force sensor we have developed, as well as a similar, previously published sensor^27^ (Figs S5 and S6) does not show any force-specific response in three *Drosophila* tissues: the wing imaginal disc, amnioserosa cells and border cells. This was unexpected because spatially and temporally varying forces have been found in these tissues by other means^15,21,22,27,33–37^ and we applied mechanical stimulations in order to exert forces on the sensor construct. Moreover, also in single molecule experiments the FRET module gave a clear response to an externally applied force^23^ and other groups have reported positive results with E-Cadherin sensors in MDCK cells and *Drosophila* border cells^26,27^. In our experiment, both for our system and for the Cai *et al*. system the qualitative behavior of the sensor and the negative control, which reveals force non–specific effects, was similar as determined by the ratiometric method as well as by FLIM. Therefore, we conclude that instrumental effects alone cannot explain the absence of a signal in our experiments. This implies that the dynamic range of the sensor or effects intrinsic to the crowded microenvironment in living tissues account for the absence of a reproducible force-specific response by the sensor module.

Before we discuss these options in detail, we will briefly consider potential problems with FRET determination and the spotlight how the lack of proper controls could be a potential reason for false positive results. This mainly concerns the wide-spread use of the ratiometric method for FRET determination^26,27,45^: the ratio of the donor and the acceptor intensity is used to calculate the FRET index. The obvious problem is that the method is intensity-based and therefore the donor/acceptor ratio depends not only depend on FRET, but on a host of experimental details in the determination of intensities, such as penetration depth, autofluorescence, laser fluctuations, microscopy settings, and etc. With respect to measuring forces, apparent differences could simply be explained by variation in intensity of the sensor or by timing differences, rather than by different tensions. For instance in our ratiometric determination of FRET, we have found a high correlation between FRET index and intensity of the signal (acceptor excitation) for border cells (r = 0.43) and wing imaginal discs (r = 0.38) (Fig. S7). While such systematic errors in the ratiometric FRET determination can give rise to false positives, this could explain the discrepancy between our results and the ones that were published previously. However, since we also did not detect a signal in FLIM experiments, which are independent of the signal intensity, systemic errors alone cannot be the reason for the absence of a signal in our experiments. To estimate the consequences of increased variation in our data, which could potentially conceal a true effect, we performed power analysis simulations. These simulations revealed that with the variations and sample size in our datasets we would be able to detect a significant change in FRET index above 5% for the wing discs and 8% for the other tissues (Fig. S8). Further, using a formerly published ATP-FRET sensor^32^, we could show in practice that despite uncertainties in the experimental setup, our methods are efficient enough to reproducibly detect changes in FRET (Fig. S1).

This sensitivity of the ratiometric FRET determination thus needs to be surpassed by the FRET signal shown by a purported FRET-based force sensor. An indication of this can be obtained from^23^, where FRET efficiencies above 10% were measured for forces below 5–6 pN on the sensor module. While the forces acting on adherens junctions in Cadherin molecules are not known directly, estimates range from 35 to 55 pN, as measured *in vitro* by AFM for the binding strength of E-Cadherin^46^, to around or below 10 pN^47,48^. While 10 pN are in the range of recently developed FRET- sensors^25^ and could be tested in further studies, 35 pN would unfold the fluorophores, thus making the estimate unlikely. These values indicate that forces on E-Cadherin might be higher than the dynamic range of our sensor. However, in our case this is not the decisive factor for the absence of a signal, because also our control construct, which does not experience a force, does not show a high enough FRET efficiency.

In addition to technical hurdles, there are possible biological or sensor-specific effects that could lead to this negative outcome in the microenvironment of a living tissue.

On the biological side, it is conceivable that E-Cadherin is either by-passed in the transduction of intercellular forces or that it is regulated in such a way that its concentration at the membrane adjusts to lead to a force homeostasis among the different Cadherin molecules – and thus tensions of single molecules would not change upon stimulation^49,50^. Our direct force changes and the corresponding time scales can test for the latter possibility to a certain extent. Given the force change due to disruption or activation of the acto-myosin network on the time scale of minutes as well as the external application of a controlled force on the same time scale would suggest that the adjustment has to be faster than this, which seems unlikely. Further, by-passing of the Cadherin molecules by other force transmitters could be likely. Given that the sensor module is much larger than the cytoplasmic domain (566aa vs. 141aa), one would expect that other force transmitters are better able to connect the cells and transmit the inter-cellular force purely by geometry. Our transgenic system, in which the sensor is expressed in the absence of the shorter, endogenous E-Cadherin, shows the same results as the previously published system, in which the sensor is expressed in addition to the endogenous E-Cadherin^27^. This argues against the possibility that the sensor molecule is by-passed by the presence of the endogenous E-Cadherin. However, other adhesion molecules or cytoskeletal structures could additionally act in parallel to the sensor molecule and transmit the mechanical load instead.

Another reason for the absence of a force-specific signal in our measurements is a bias of FRET due to the densely crowded microenvironment in the living tissues. One option for such a quenching is intermolecular FRET due to the close proximity of E-Cadherin molecules at the membrane. To test for this, we have looked at flies expressing E-Cadherin with only YFP and only CFP in parallel. Because we did not observe FRET above background noise in these flies, we can exclude intermolecular FRET in our experiments. Another possible reason for the lack of a force-specific signal could be the inefficient maturation of fluorophores. Differences in maturation efficiency between the donor and the acceptor strongly bias the resulting FRET efficiency and would further lower the sensitivity of the FRET measurements. While it is very difficult to control for the maturation efficiency, our observation that the two sensors, which comprise different fluorophores, display comparable FRET efficiencies, argues against maturation efficiency being the main problem in our analysis. The remaining property for a biased FRET is that of orientational mismatch of the fluorophores which reduces FRET^51^. Such an orientational mismatch can be due to the large size of the sensor compared to the cytoplasmic domain of E-Cadherin and would be present for both the sensor and the control constructs in equal measure. While orientational effects are present in all FRET sensors, this is usually seen as a random dynamic process and can be averaged out^52,53^. Because our sensor is monomolecular, meaning that the fluorophores are linked together, the relative orientation is biased and not random any more. In this case, it has been shown that apart from the distance also the orientation has a significant impact on FRET efficiency^53^, comparable to the impact of the distance on FRET^24^. So far, we can only speculate about the orientation of our FRET sensor *in vivo*, but it is very likely that conformation of E-Cadherin, or neighboring proteins, affect the relative orientation of the FRET pair. This could however explain the difference in FRET between shgContr and shgFRET and could also explain why similar changes in FRET occurred with shgContr and shgFRET in some experiments. Finally, it is well known that FRET efficiency is also sensitive to pH as well as the refractive index of the medium surrounding the FRET pair^51,54^. This could partially explain why FRET changes upon *in vitro* culturing or the application of an osmotic shock, which both affect the intracellular environment (Fig. S9).

In conclusion, our *in vivo* approach to develop a FRET based tension sensor revealed several technical challenges. Even though we used a revised version of previously published E-Cadherin tension sensors, we did not observe any sign that our sensor, as well as an already published sensor, could reproducibly measure forces in the three different *Drosophila* tissues. This study highlights general problems and potential pitfalls with the analysis and interpretation of FRET based tension sensors and will hopefully spur follow-up projects to overcome these difficulties.

## Materials and Methods

### Drosophila strains

Fly stocks were grown on a standard cornmeal medium at 25 °C. Cad^Contr^, Cad^TS^, Cad-Venus and Cad-mTFP (Bloomington #58368, #58365, #58367, #58366) (a gift from D. Montell) were used in experiments analogous to shgContr, shgFRET, shgYFP and shgCFP. Border cell-specific *slbo*Gal4 driver and UAS-*lifeact*-RFP (Bloomington #58435, #58362) were used to label the border cell cluster for segmentation. AT1.03RK2 and AT1.03NL2 (DGRC #117014, #117012) with the driver lines *salE*Gal4 (Denise Nellen, FBrf0211371, 4.8 kbp EcoRI fragment 2 L:11459156..11454345 Dmel_r6.08 generated in our laboratory) and *slbo*Gal4 were used for experiments with ATP-FRET. For live movies, *sqh*-GFP, *moesin*-GFP, and DE-Cad*-GFP* (Bloomington #57144,^55,56^) were used.

### Generation of transgenic flies

shgFRET, shgContr, shgYFP and shgCFP were generated by a knock-in of the sensor module into the endogenous locus of E-cadherin (*shotgun*), as previously described^56^. We used the sensor module published by Borghi *et al*.^26^ but with the mTFP1 exchanged by an ECFP and a Gly Ser rich flexible linker: GSGGTGSTSGGSGGSTGG (gifts from Alex Dunn). For integration we used the plasmid DE-Cad^(rescue)^ from Huang *et al*., a pGE-attB- vector containing a fragment of *Drosophila* E-cadherin. For shgFRET, shgCFP and shgYFP we introduced the restriction sites KpnI and SphI into the cytoplasmic domain of E-Cadherin, between the *p120*- binding site and the transmembrane domain, after amino acid G1356 of E-Cadherin. Following primers were used: CGGGGTACCTGGCACGAAAAGGACATCGA (KpnI) and ACATGCATGCGCCATTCTTCTGCTTTTTCT (SphI).

We inserted the FRET sensor (shgFRET), only ECPF (shgCFP) or only EYFP (shgYFP) via the restriction sites for SphI and KpnI. Following primer pairs, flanked by a KpnI or SphI, were used for amplification:

shgFRET: ACATGCATGCGGATCAGGTGGAACTGGTT and CGGGGTACCACCTCCTGTTGAACCTCC

shgCFP: ACATGCATGCGGATCAGGTGGAACTGGTT and CGGGGTACCGAACAGCTCCTCGCCCTT

shgYFP: ACATGCATGCGACGAGCTGTACAAGTTA and CGGGGTACCACCTCCTGTTGAACCTCC

For shgContr we introduced KpnI and SphI before the STOP codon, with the primers CGGGGTACCTAGGAATCTTCGCCAGCC (KpnI) and ACATGCATGCGATGCGCCAGCCCTGGTCAT (SphI). The same amplicon as for shgFRET was inserted by KpnI and SphI.

These constructs, cloned into the DE-CAD^(rescue)^ vector, were microinjected into the founder line DE-CadGX23w[-]/CyO^56^. Microinjection was performed by the Huazhen Biotech Company.

### Immunohistochemistry

Immunostaining of the wing imaginal disc was performed according to standard protocol. Primary antibody anti-armadillo (AB_528089, Developmental studies hybridoma bank) and secondary antibody goat anti-mouse Alexa Fluor 594 (Molecular Probes, 1:500) were used.

### Live imaging

Wing discs and salivary glands were dissected from 3^rd^ instar larvae in WM1, mounted in a glass bottom dish (Imaging dish CG, Bioswisstec) covered with a cell culture insert (Millipore), as previously described^57^. Because timing of dissection and imaging was critical, we dissected shgContr and shgFRET alternating to have best control for timing effects.

To image border cell migration, egg chambers were dissected in Schneider’s medium (Invitrogen) supplemented with 15%FBS (Gibco) and 200 mg/ml insulin (Sigma-Aldrich) from 3–4 days old, well fed female flies. Egg chambers were mounted in a poly-L-lysine (Sigma-Aldrich) coated glass bottom dish (Imaging dish CG, Bioswisstec). (Adapted from^58^, Methods in Mol Biol).

For dorsal closure, embryos were aged for around 18–20 h at 25 °C, dechorionated in 50% bleach and mounted in Voltalef 10 s oil (VWR) on cover slips (Menzel Gläser).(Adapted from^59^).

Images were acquired with a Zeiss LSM710 microscope with an Argon laser, if not otherwise stated.

Movies were taken with an Andor revolution spinning disc confocal microscope and an Andor iXon3 EMCCD-camera.

### Pharmacological treatment

For pharmacological treatments the drugs were directly added to the culture medium for wing disc, salivary glands or border cell migration. To inhibit actin polymerization in the wing disc, Latrunculin B was added to the WM1 (10 µM, Sigma Aldrich) and imaged 5 after. To increase cell volume of the wing disc by an osmotic shock, distilled H_2_O was added to a final concentration of 50% and imaged 5 minutes after. To decrease Myosin activity in the border cells, the ROCK-inhibitor Y-27632 was applied (100 µM, Sigma-Aldrich) and images were taken 30–45 min after. To modify the activity of the ATP-FRET sensor, Antimycin A (20 µM, Santa Cruz Biotech) was added to the culture of wing disc, salivary gland and egg chamber and imaged as indicated in the figures.

### Stretching device

In order to apply an external force to the cultured wing disc, we used the stretching device as described previously^60^. The wing pouch of the dissected wing disc was attached to a glass slide, whereas the notum was attached to a small, moveable cover slip. Poly-L-lysine (Sigma Aldrich) was used for adhesion. The moveable cover slip was attached to a spring sheet which we used to apply a calibrated force to the disc. The force was calculated with the formulae adopted from the equation for the spring constant of a cantilever (*L* is the length, *a* the thickness, *b* the width and *E* the elastic modulus of the spring sheet, d is the distance that the spring sheet is displaced):

1 $$F=-\,\frac{E\,\ast \,{a}^{3}\,\ast \,b}{4\,\ast \,{L}^{3}}d$$

For measuring the effect of an applied force, we alternated between a pre-stretched state (10 µN), to pull the disc until it was taut, and a stretched state (25 µN).

### FRET analysis

#### Sensitized emission

For FRET analysis images were taken with the Zeiss LSM710 in three different channels: (1) YFP: 514 nm laser; filter: 525–570 (2) CFP: 458 nm laser; filter: 463–505 nm (3) FRET: 458 nm laser; filter 525 nm-570. To correct for the crosstalk between the channels due to spectral overlap, we calculated the sensitized emission (SE)^31,61^. By bleed-through, we infer here the leak-through of CFP signal into the YFP detector. By cross-excitation we refer to the direct excitation of YFP with the 458 nm laser. To correct for the bleed-through we used the shgCFP flies and calculated the correction factor α = I^FRET^/I^CFP^(I^FRET^ = intensity FRET channel; I^CFP^ = intensity CFP channel; I^YFP^ = intensity YFP channel). To correct for cross-excitation we used the shgYFP flies and calculated the correction factor β = I^FRET^/I^YFP^. Depending on the tissue and the microscopy settings, the values for α and β varied between 0.05–0.15. With these factors we obtained the SE from shgFRET and shgContr by:

2 $$SE={I}^{FRET}-\alpha \,\ast \,{I}^{CFP}-\beta \,\ast \,{I}^{YFP}$$

The FRET index was further calculated by the ratio:

3 $$FRET\,index=SE/({I}^{CFP}+SE)$$

#### Image analysis

Fluorescent images were analyzed with Fiji, a distribution of ImageJ, using in-house macros.

Raw images were blurred with a median filter (sigma = 1), oversaturated pixels were removed and background was subtracted using the rolling ball algorithm. Further, the image stack was projected by a maximum-intensity z-projection and masked with an automated threshold from CFP and YFP channels (Otsu algorithm). Subsequently, the FRET index was calculated pixel by pixel as described above. Finally, negative pixels were deleted and the look up table “Fire” was applied for visualization of the results. For an overall FRET index of one image, the mean of the masked image was taken.

#### Image segmentation

To analyze cell size, cell orientation and edge length of the wing disc and the amnioserosa, we processed the images in the YFP-channel by using FIJI and Epitools (Icy plug-inn,^62^). First, images were blurred and background subtracted as described above, then local maxima were determined and particles segmented to obtain a segmented binary image in FIJI (*Find Maxima – Segmented Particles*). Second, the segmented binary images and the calculated FRET images were overlaid with Epitools (*CellGraph* and *CellOverlay*) and the values for FRET indices combined with cell size, edge length and orientation extracted.

To analyze amnioserosa cells, we either distinguished between cells that contract/expand between 7 minutes (1) or cell edges that contract/relax within 1 minute (2). (1) To determine cells according to their size, we took high quality image stacks at time-point 0 min and 7 min to calculate the FRET index. Every minute in between (time-points 1, 2, 3, 4, 5, 6 min) we took snapshots to determine the cell area. We defined time-points 0 and 7 to be in a different pulsing stage (contracted vs. expanded) if they differ in cell area for more than 10% and they differ in more than one standard deviation, calculated from all the time-points together. (2) To determine edges according to their length, we measured length as the distance between two vertices. We took two high quality image stacks within one minute and distinguished contracted and expanded edges if they differ for more than 20% in length.

To analyze border cells of Cad^TS^ and Cad^Contr^ ROIs, covering on average 20 µm^2^ of masked image, in the front and the back were chosen according to the channel for *slboG4::UAS-lifeact-RFP* and the information about orientation from an overall image. For shgFRET and shgContr the YFP channel was used instead of *slboG4::lifeact-RFP*.

#### Intermolecular FRET

To test for the occurrence of intermolecular FRET between neighboring molecules, we compared FRET indices from wing discs with either (1) one copy of shgFRET, (2) one copy of shgCFP or (3) one copy of shgCFP and shgYFP expressed in parallel.

### FLIM

#### Image acquisition

Images were taken with a Leica SP8 confocal microscope covering a TCSPC-FLIM module from Picoquant (PicoHarp300) and the SymPho Time 64 software. For shgFRET and shgContr, a pulsed diode laser (PDL 800-B) (440 nm, 40 MHz) and a HyD SMD detector (450–505 nm) were used. For Cad^TS^ and Cad^Contr^ a White Light Laser (at 470 nm, 40 MHz) and a HyD SMD detector (480–505 nm) were used. (Imaging performed at ScopeM –Image facility at ETH Zürich).

Mounting and imaging was performed as described above for the Zeiss LSM710.

#### Image analysis

Lifetime data were analyzed using the SymPho Time 64 software. For an overall lifetime value of one image, we fitted a double-exponential, reconvolution (calculated IRF) model to the lifetime histogram of the image and used the intensity weighted lifetime (τ ^Av Int^). For spatial patterns of lifetime across the wing disc, we set a binning of 2 × 2 pixels and a threshold to remove the background and calculated a FLIM Fit. To calculate FRET efficiency €, we took lifetimes from donor only (τ^shgCFP^) and the FRET pairs (τ^shgFRET^ or τ^shgContr^):

4 $${\rm{E}}=1-{{\rm{\tau }}}^{{\rm{shgFRET}}}/{{\rm{\tau }}}^{{\rm{shgCFP}}}$$

For Cad^TS^ and Cad^Contr^ analysis was done accordingly.

### Statistics

Statistics were performed in R. Significance was calculated by with Welch’s t-test, which assumes unpaired samples with unequal variance. Significance levels were indicated as ***(p ≤ 0.001), **(p ≤ 0.01), *(p ≤ 0.05) and n.s. (p > 0.05). To estimate the correlation between two samples, the Pearson’s correlation coefficient R was calculated.

To estimate the *minimal difference* between the means which would theoretically be detectable with our data, we performed a power analysis (significance level = 0.05, power = 0.8). Therefore, we took a dataset of shgContr, randomly picked two samples and calculated the *effect size* and *standard deviation*. Then we randomly picked three new samples and again calculated the *effect size* and *standard deviation*. This was repeated until we reached the sample size of the entire data set. These permutation assays were repeated 10.000 times. Then, the average *effect size* from the 10.000 permutations for each sample size was calculated. We obtained the *minimal detectable difference* from the *effect size* d:

5 $$d=\frac{\mu 1-\mu 2}{\sigma }$$

µ1 and µ2 are the means and σ the standard deviation. The *minimal detectable difference* is the difference between the means.

## Electronic supplementary material

Supplementary Info
