# Supplementary material for: Challenging FRET-based E-Cadherin force measurements in Drosophila
Source: Sci Rep. 2017 Oct 20;7:13692. doi: 10.1038/s41598-017-14136-y (PMC5651909; doi:10.1038/s41598-017-14136-y)
Supplement: Supplementary file 1 — Supplementary Info [file 41598_2017_14136_MOESM1_ESM.pdf]

Challenging FRET-based E-Cadherin force measurements in *Drosophila*

Dominik Eder[1,2], Konrad Basler[1], Christof M. Aegerter[1,2]

[1] Institute of Molecular Life Sciences, University of Zurich, CH-8057, Switzerland.

[2] Institute of Physics, University of Zurich, CH-8057, Switzerland.

Correspondence to CM. A (aegerter@physik.uzh.ch)

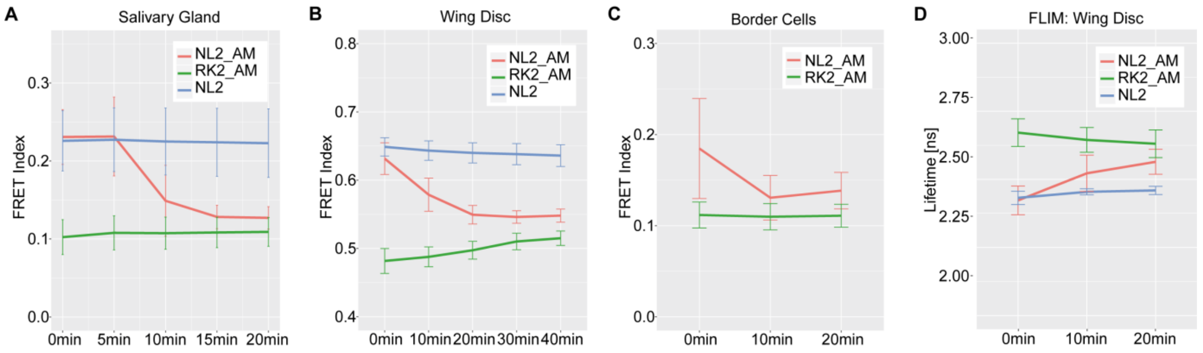

Figure S1. FRET analysis of ATP sensor as positive control. We used an established FRET sensor to test our analysis workflow. This ATP (Adenosine triphosphate) activity sensor changes conformation from an open state to a closed state by binding of ATP (Tsuyama et al., 2013). Hence, an increased FRET index indicates high abundance of ATP. A functional sensor (ATP-NL2) and an insensitive version as negative control (ATP-RL2) were used. We treated the samples with the chemical AntimycinA (AM) to decrease ATP levels. Using the ratiometic method, the sensor was applied in the salivary gland (A), as in the original publication, in the wing disc (B) and in the border cells (C). In all three tissues the FRET index decreased upon AM treatment as expected within 5- 20 minutes. The negative control RK2 was not affected or slightly increased by the treatment. (D) Similar results were obtained when FRET was measured with FLIM. These results confirmed that our FRET analysis pipelines are sensitive to FRET.

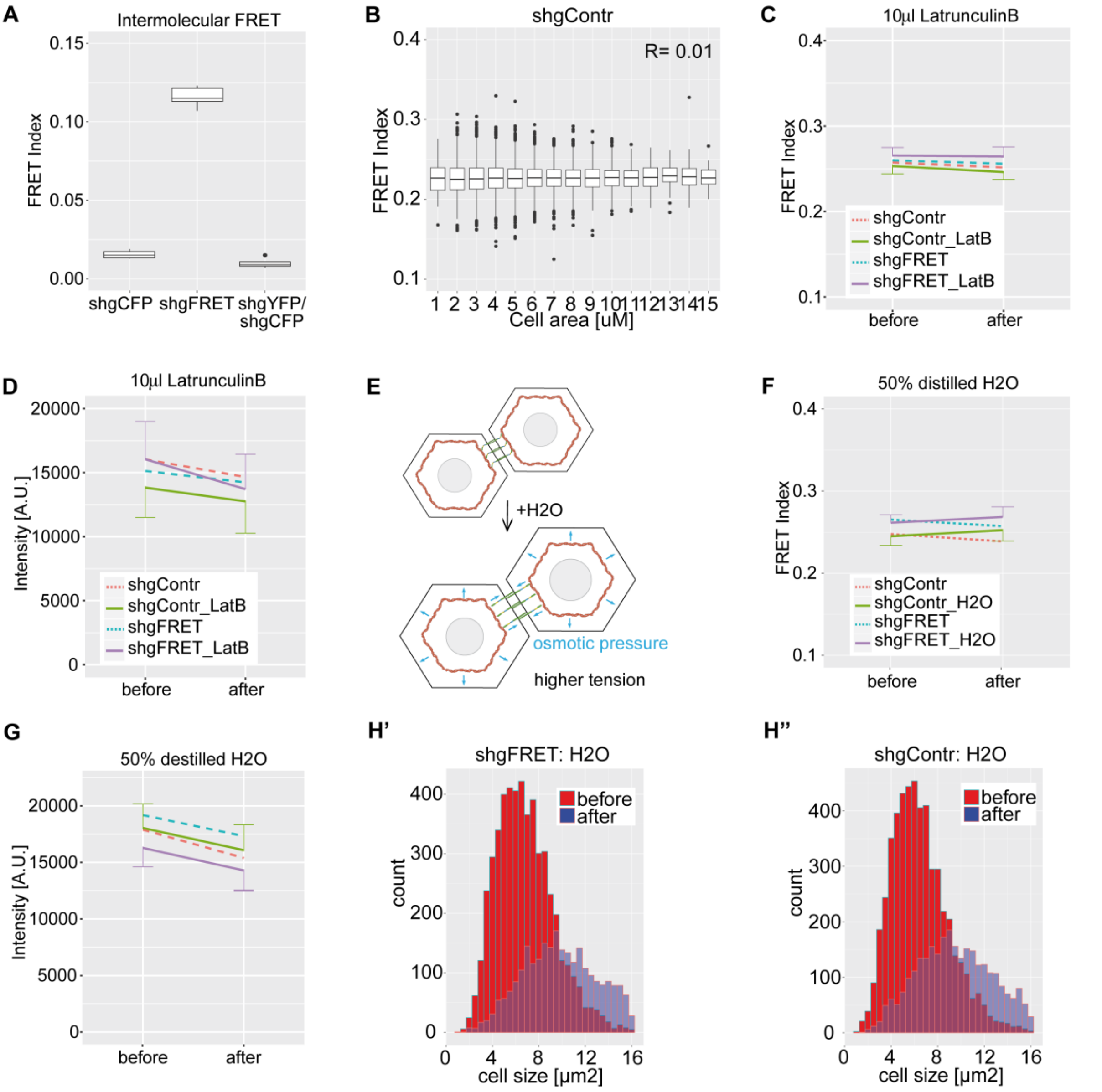

Figure S2. FRET analysis in the wing disc. (A) Test for intermolecular FRET: FRET index for wing discs with shgYFP and shgCFP expressed in parallel are similarly low (0.010, n=6) as the background in shgCFP (n=0.015,n=6) , and around 10x lower than in shgFRET (0.116, n=7). (B) The FRET index of individual cells did not correlate with cell area in the wing pouch (here shown for shgContr, data pooled from 14 wing discs; n>21.000 cells). (C) FRET index decreased for shgContr (2.7%, p=0.026, n=18) and shgFRET(0.4%, p=0.74, n=18) upon treatment with LatrunculinB within 5 minutes. But also without treatment (dashed lines) the FRET index decreased over time for shgContr (2.2%, p=0.014, n=9) and shgFRET (1.5%, p=0.19, n=9). (D) In the same experiment as in (C), also the intensity of the YFP channel decreased for shgFRET and shgContr, with (14%, p=0.018 vs. 7.7%, p=0.189) and without (5.9% p= 0.415 vs. 8.6%, p=0.013) treatment. (E) The treatment with distilled H2O causes an osmotic shock and increases the cell volume. We expect that the distance between the acto-myosin rings of adjacent cells increases and thereby stretches E-Cadherin. This would result in a decrease in FRET index. (F) But instead of an expected decrease, the FRET index of both shgFRET (2.7%, p=0.03, n=24) and shgContr (3.0%, p=0.04, n=24) increased. The control without treatment (dashed line, n=12) decreased over the 5 minutes of experiment (3.0%, p=0.06 vs. 3.6%, p=0.12, n=12). (G) In the same experiment as in (F), the intensity of shgFRET and shgContr decreased, with

(10%, p=0.003 vs. 12%, p=0.0003, n=24) and without (9.6%, p=0.6, vs. 13%, p=0.001, n=13) treatment. (H', H'') Upon $H2O$ treatment, the apical cell area increased for around 40% for shgFRET and shgContr.

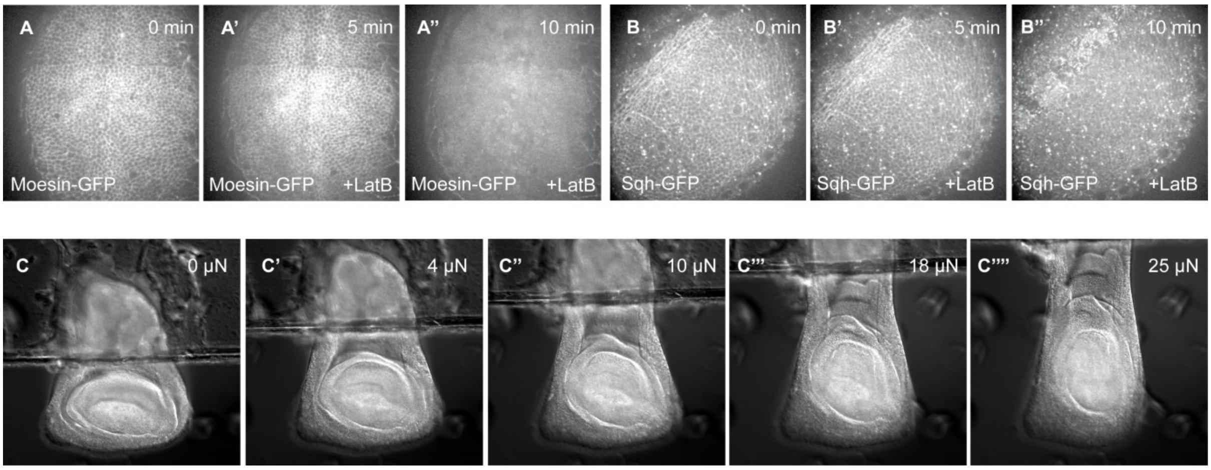

Figure S3. Mechanical stimulations in the wing disc. (A, B) LatrunculinB treatment is supposed to inhibit actin polymerization and thereby decreases the cortical tension. To test the efficiency and speed of our treatment, we analyzed the dynamics of the actin-binding protein Moesin (A, A', A'') and the *Drosophila* homolog of Myosin, Spaghetti squash (sqh) (B, B', B''). For both, the intensity of the signal dropped and the signal disappeared from the membranes within the first 10 minutes. (C-C'''') Here, we show an example of a wing disc which was stretched with an increasing force. Images show the transmission light channel. (C'') and (C'''') represent the applied forces which were used for the experiment, 10µN and 25 µN (Fig. 2).

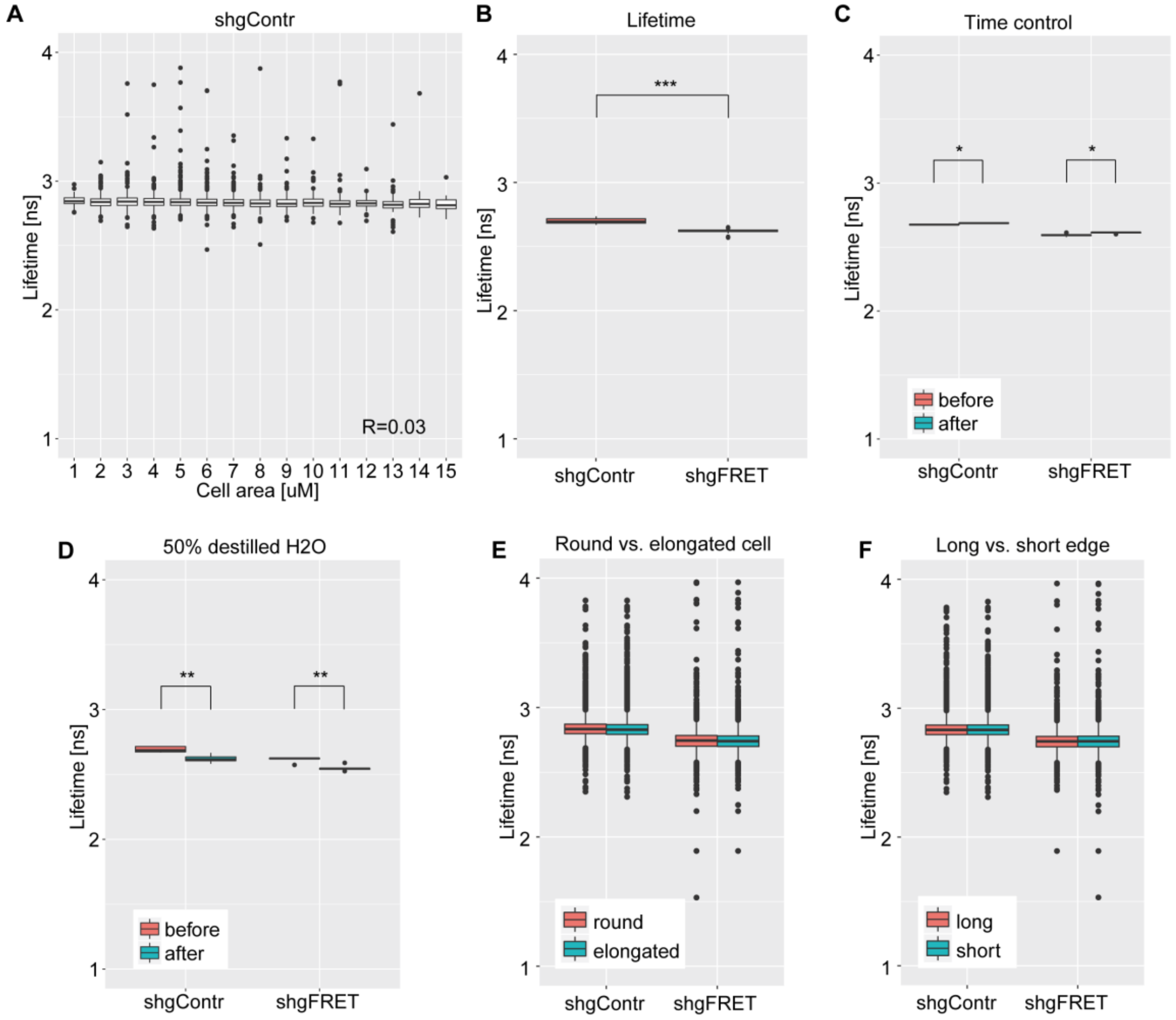

Figure S4. FLIM measurements in the wing disc. (A) The fluorescence lifetimes of individual cells did not correlate with cell area in the wing pouch (here shown for shgContr, pooled from 8 wing discs, n>5000 cells). (B) Lifetimes of the entire wing pouch were significantly higher for shgContr (2.7, n=15) than for shgFRET (2.6, n=14). (C) The lifetimes of shgContr (2.68 vs. 2.69, n=4) and shgFRET (2.59 vs. 2.61, n=4) increased over the 5 minutes of the experiment, even without any treatment. (D) The application of an osmotic shock by adding distilled H2O decreased the lifetimes for shgContr (2.69 vs. 2.62, n=5) and shgFRET (2.61 vs. 2.55, n=9). (E) Lifetimes did not differ between round vs. elongates cells for shgContr (2.83 vs. 2.83) and shgFRET (2.74 vs. 2.74), neither between long vs. short edges (F) for shgContr (2.83 vs. 2.83) and shgFRET (2.78 vs. 2.78). (Data for A, E and F were pooled from 8 wing discs, n>13.000 cells)

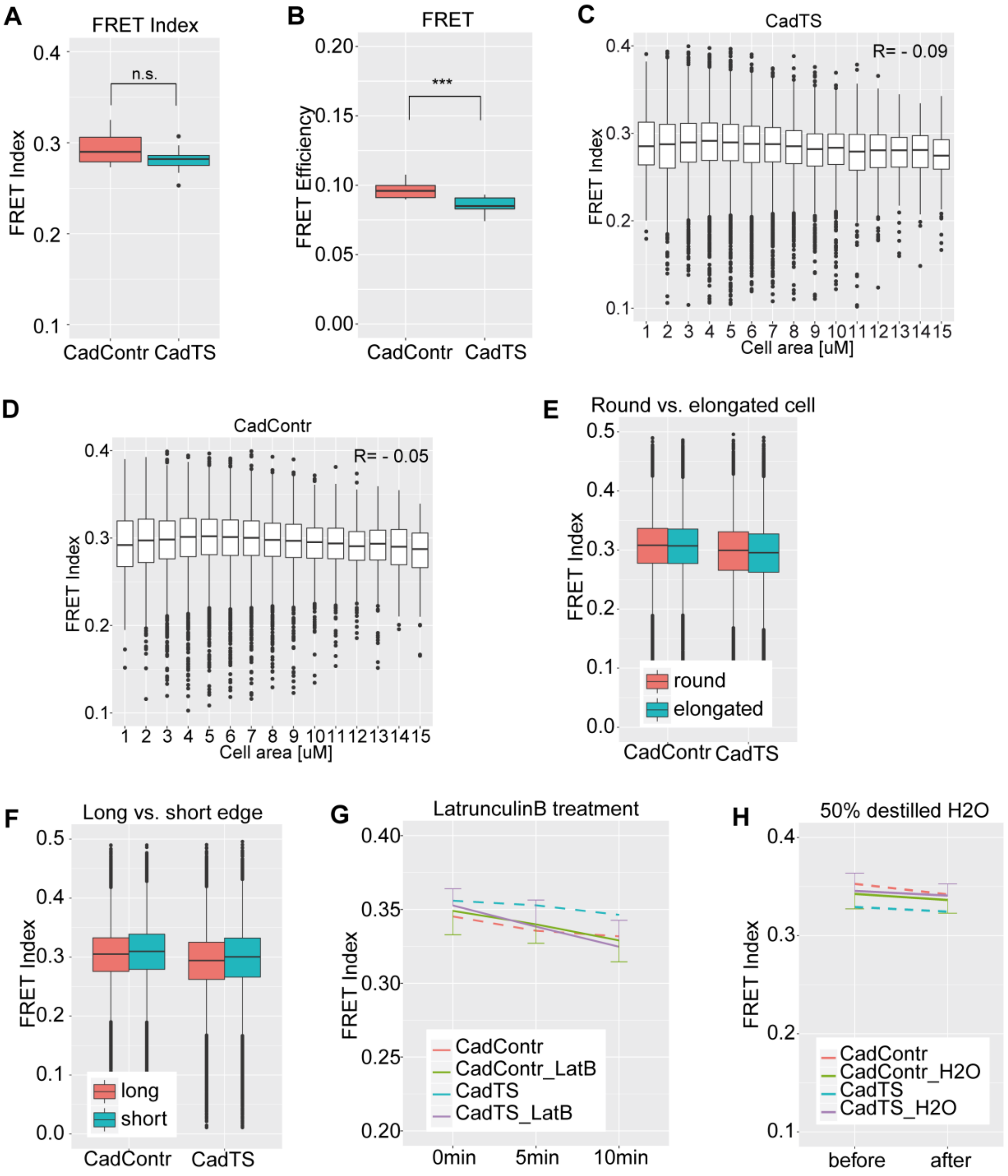

Figure S5. FRET analysis of CadTS sensor in the wing disc. (A) The FRET index in the entire wing pouch for CadContr (0.292, n=13) is 4% higher than for CadTS (0.281, n=13). (B) The FRET efficiencies of wing discs , measured by FLIM, are 0.096 (n=10) for CadContr and 0.085 (n=10) for CadTS (C, D) For both, CadTS and CadContr, the FRET index of individual cells does not correlate with cell size. (E) The FRET index of individual cells is similar between round and elongated cells for CadContr (30.6 vs. 30.5), but they differ around 1.5% for CadTS (29.7 vs. 29.3). (F) The FRET index of an individual edge is around 1.5% lower for long than for short edges, for CadContr (30.2 vs. 30.7) and CadTS (29.2 vs. 29.7). (Data for C-F were pooled from 13 wing discs, n> 20.000 cells) (G) After LatrunculinB treatment, the FRET index for CadContr and CadTS decreases slightly more than for the time control without treatment (dashed line). (H) After treatment with distilled H2O, the FRET index

decreases for CadContr and CadTS slightly less than for the time controls (dashed line). ((G, H) n=18 for treated samples and n=9 for time controls)

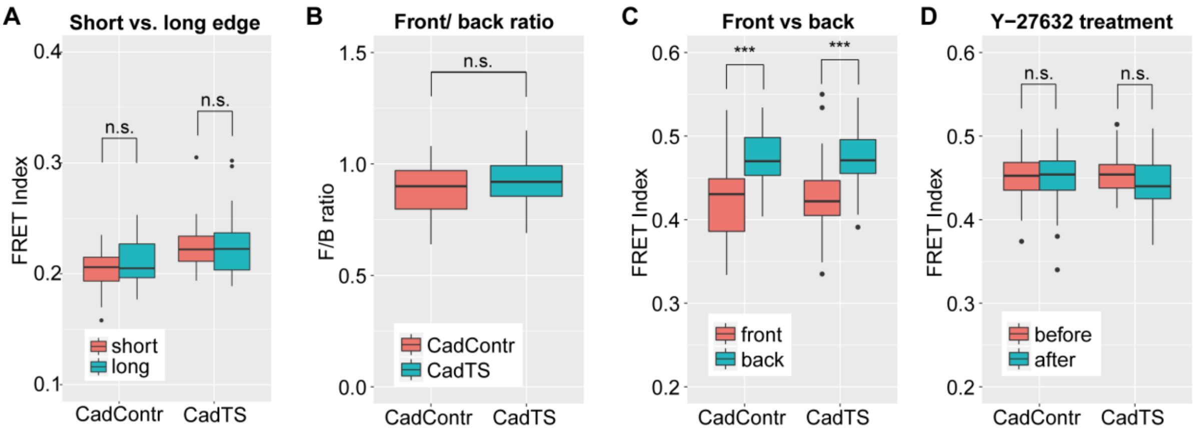

Figure S6. FRET analysis of CadTS in the amnioserosa cells and in the border cells. (A) Cells which during dorsal closure either elongate or shorten within one minute do not change their FRET index. (CadContr n=55, CadTS n=44). (B, C) Both CadContr (0.89) and CadTS (0.92) have a front to back ratio below one, which shows that the FRET index is higher in the back than in the front of the border cell cluster. But CadContr and CadTS do not significantly differ in their front to back ratio. (D) Myosin downregulation by Y-27632 treatment does not significantly change the FRET index for CadContr and CadTS. ((B, C, D) n=26 for CadContr and n=30 for CadTS)

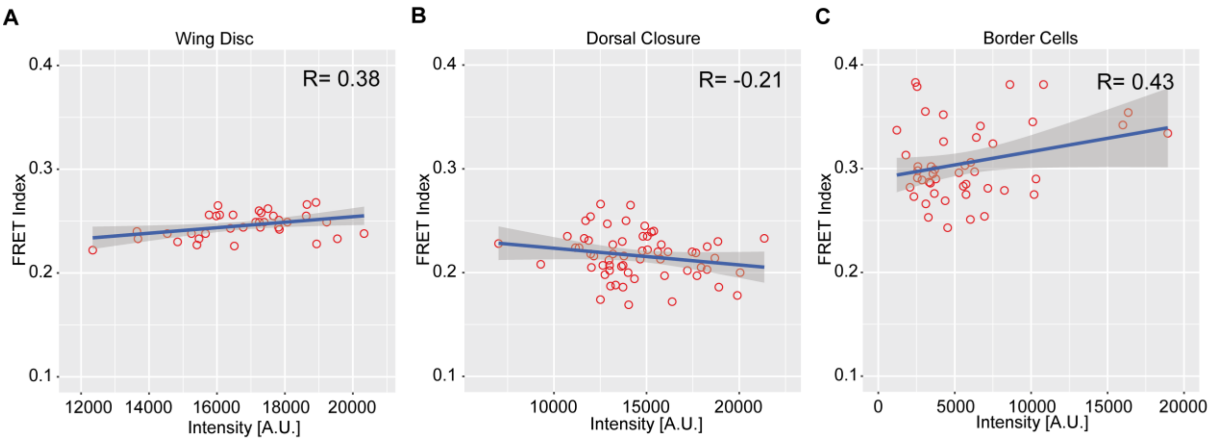

Figure S7. Correlation between FRET index and acceptor intensity. For datasets of shgContr flies without treatment or time dynamics, there is a moderate linear correlation between the FRET index and the intensity in the YFP channel for (A) the wing discs (n=36) and (C) border cells (n=48). For dorsal closure, there is no linear correlation (n=62), only a slight tendency that FRET index decreases with increasing intensity. The blue line shows a linear regression model with the confidence interval in dark grey.

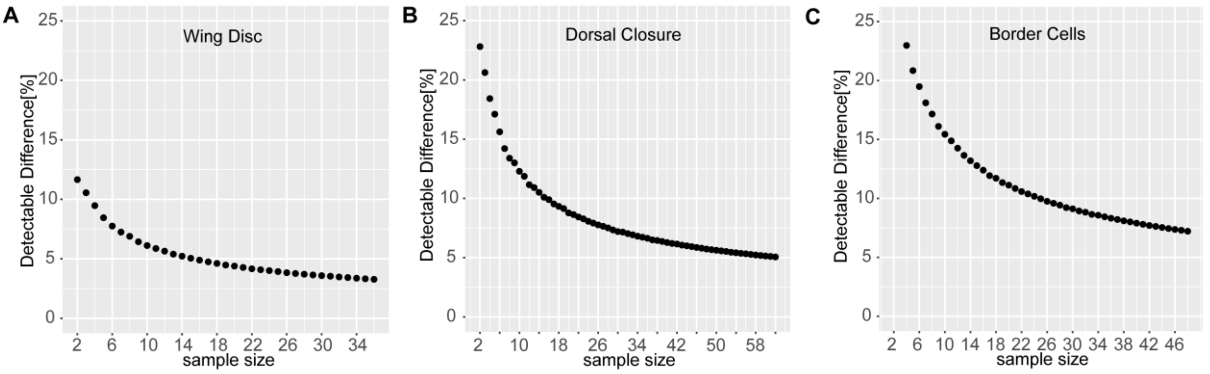

Figure S8. Power analysis simulations to estimate the minimal detectable difference. For three different datasets of shgContr: wing disc (A), dorsal closure (B) and border cell migration (C) we performed power analysis to estimate the minimal effect that we would detect with our data – with a given significance level of 0.05 and a statistical power of 0.8. The graphs indicate the minimal difference between two means that we would theoretically detect as a function of the sample size. E.g. with a sample size of 18 wing discs we would be able to detect a minimal significant effect that changes the FRET index for 5%.

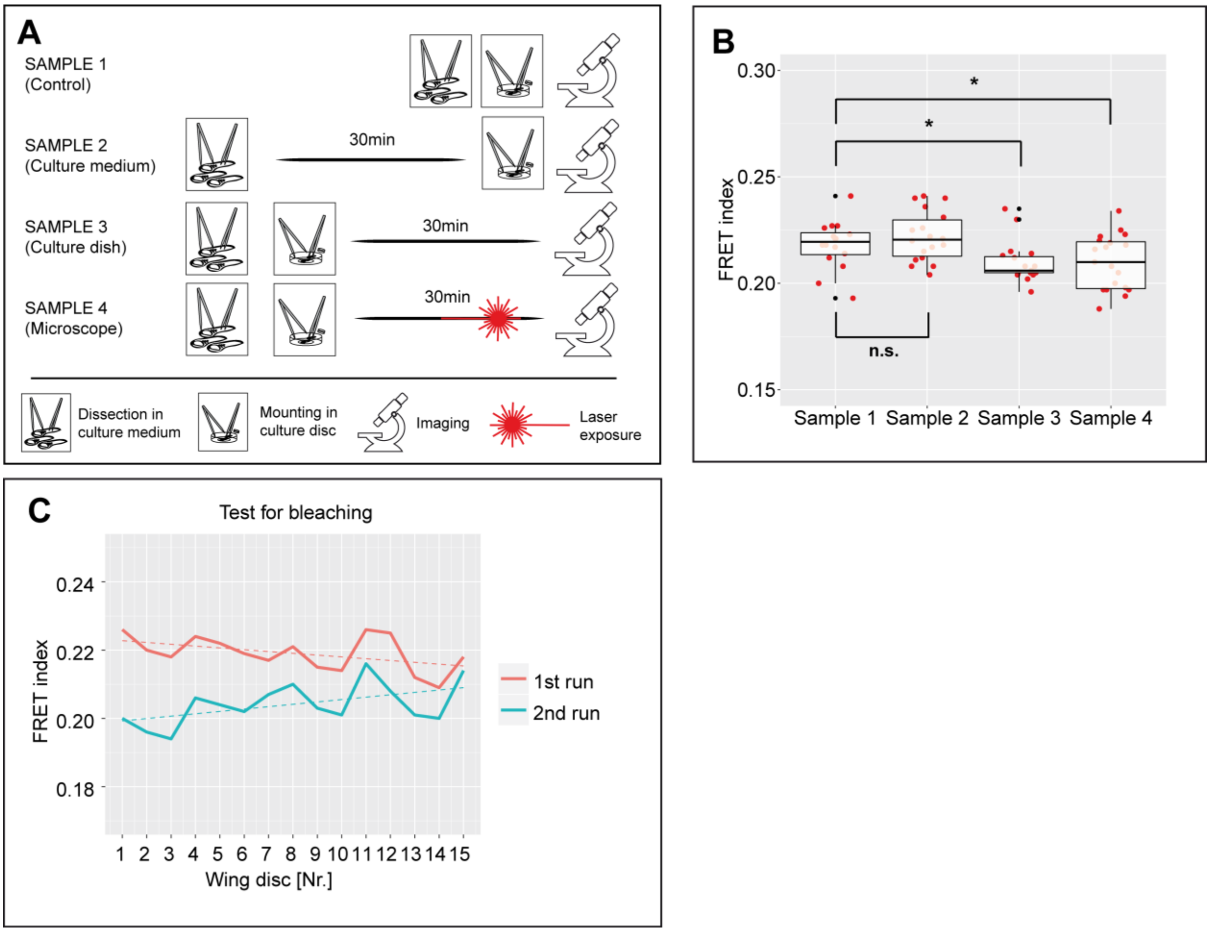

Figure S9. Analysis of the decrease in FRET index over time under culture conditions. (A) Schematic representation of the experiments to test whether the FRET index is temporally affected by the culture medium (sample 2), the mounting of the discs in the imaging chamber (sample 3) or the process of imaging at the microscope (sample 4). In sample 1, wing discs were dissected, mounted in the imaging chamber and imaged directly. In sample 2, wing discs were cultured in culture medium for 30 minutes before mounting. In sample 3, wing discs were kept in the imaging chamber for 30

minutes before imaging. In sample 4, the imaging chamber was kept at the microscope table with laser exposure (not directly at the wing discs) for 30 minutes. (B) Dissecting and culturing the wing discs in medium did not affect the FRET index over time, but mounting the wing disc in the imaging chamber did. Exposure of the imaging chamber to the laser (e.g. heating of the medium) did not further affect the FRET index over time. (C) To test the effect of bleaching, 15 wing discs were imaged consecutively for 30 minutes in a first run. In a second run, the wing discs were imaged again in reverse order. If photo-bleaching took place, the two regression lines would be parallel. But the regression lines show a constant decrease of FRET index over time.
